# Oak flour as a replacement of wheat and corn flour to improve biscuit antioxidant activity

**DOI:** 10.1002/fsn3.524

**Published:** 2018-01-12

**Authors:** Mitra Parsaei, Mohammad Goli, Hajar Abbasi

**Affiliations:** ^1^ Department of Food Science and Technology Isfahan (Khorasgan) Branch Islamic Azad University Isfahan Iran

**Keywords:** antioxidant activity, biscuit, oak flour replacement, peroxide value, sensory evaluation

## Abstract

Due to the high antioxidant activity of oak fruits, the partial substitution effects of wheat flour (WF) or corn flour (CF) with oak flour (OF) have been investigated. WF or CF was replaced by OF at levels of 0%, 15%, 30%, and 45% in the biscuit formulations to prevent the spontaneous oxidation of lipids, and the characteristics, including peroxide value, antioxidant activity, and sensory properties, were evaluated during 28 days storage. According to the results obtained, biscuit samples with higher OF percentage had higher antioxidant activity and less peroxide value. In terms of sensory evaluation, no adverse effect was observed in the samples containing OF. Therefore, it could be introduced as a good natural source of antioxidant compounds for use in food formulations.

## 1 INTRODUCTION

Biscuits are the most popular bakery products consumed by all levels of society, mainly due to its ready‐to‐eat nature, high nutritional quality, availability in different varieties, and affordability (Sudha, Vetrimani, & Leelavathi, 2007). However, this product has considerably high amounts of lipid (Mildner‐Szkudlarz, Zawirska‐Wojtasiak, Obuchowski, & Gośliński, 2009), which oxidizes slowly during storage (Reddy, Urooj, & Kumar, 2005). Lipid oxidation leads to undesirable flavor in biscuits. The loss of color and nutrient value, and more importantly, the accumulation of deleterious compounds are the other results of this chemical reaction (Mildner‐Szkudlarz et al., 2009). Therefore, maintaining the high quality of biscuits is of great nutritional and economic importance, since this product is widely used and often stored for extended periods before consumption (Mildner‐Szkudlarz et al., 2009; Reddy et al., 2005). Lipid oxidation may be prevented by the use of oxidation inhibitors or antioxidants. Synthetic antioxidants have been used in food since the beginning of the last century; however, their use has begun to be restricted because of their toxicity (Byrd, 2001; Reddy et al., 2005). Recently, natural plants have received much attention as sources of biologically active substances, including antioxidants, antimutagens, and anticarcinogens (Dillard & German, 2000). Acorns, the fruit of oak trees, have been an important part of traditional diets of people throughout the world and are reported to have potential health benefits (Polimac, Komlenic, & Lukinac, 2015). Acorn flour is desirable from a nutritional point of view, because of the content of fat (of which over 80% is unsaturated), proteins, and considerable amount of electrolytes (calcium, magnesium, potassium, and phosphorus), but little or no sodium, and is rich in iron, copper, and zinc. Acorn meal could be a nutritionally functional ingredient in foods that use wheat flour such as cookies, muffins, breads, noodles, pastries, and deserts with a growing presence in the food industry to improve the eating habits of individual clients and the general population for health benefits and disease prevention (Polimac & Komlenic, 2015). Aside from nutritional properties, oak fruits, acorn, contain phenolic compounds such as the derivatives of benzoic acid, cinnamic acid, proanthocyanidins, quinones, flavons, chalkons, and amino phenolic compounds with high antioxidant activity, which can restrain free radicals, break the chain of radical reactions, and entrap the metals. Increasing the consumption of these compounds leads to a decrease in the risk of digestive diseases and dangerous cancers (Bonoli, Marconi, & Caboni, 2004; Cantos et al., 2003; Muchuweti, Ndhlala, & Kasiamhuru, 2006). The present study is an attempt to evaluate the effect of oak flour (OF) utilization as a source of natural antioxidants on the stability of fat in biscuits. OF was used in replacement of wheat (WF) or corn flour (CF), and its effects were investigated.

## 2 MATERIALS AND METHODS

### 2.1 Materials

The oak fruits (*Quercus brantii*) were collected from the forests of Fars province in Iran. WF and shortening were purchased from the Tarkhineh Karaj Co. and the Kamoor Co., respectively, in Iran. Sugar, sodium carbonate, eggs, and invert syrup were bought from the local market.

### 2.2 Oak flour preparation

The wooden shell of oak fruits was removed and dried in an ambient condition for 28 days. The inner shell (placenta) of the fruit was separated and pounded by a hammer mill. Finally, OF was produced by sieving (mesh size = 20).

### 2.3 Biscuit preparation

The biscuit dough was produced according to Table 1. In order to evaluate the effect of OF on biscuits properties, this flour was used as a replacement for WF or CF, according to the formulations given in Table 2. All ingredients were mixed for 5 min in a mixer (Major Classic KM636, Kenwood, UK), at the second speed stage. Then, the biscuit dough was rolled out to a thickness of 5.5 mm and cut into a diameter of 5 cm. The biscuits were baked for 15 min at a temperature of 250°C of the oven (Imen Gas, Iran). Biscuits were baked for 15 min, cooled in ambient temperature (20–25°C), and stored in polyethylene packages until the testing time.

**Table 1:** Formulation and stages of biscuit production

Step	Raw materials	Amount (%, on the basis of the weight of flour)	Method
1	Shortening Sugar	22.32 12.17	Mixing to create the raw mode/cream (about 8 min)
2	NaCl Egg yolk Water Invert syrup	1.6 3 2 12.17	Adding to the mixture and stirring for 10 min at high speed
3	Sodium bicarbonate	2	Adding to the flour and sieving with the flour twice
4	Flour	100	Adding and mixing with the other components to obtain a good quality dough

**Table 2:** Biscuit samples formulations and their codes

Formulation	Code
100% CF	C/100
15% OF + 85% CF	C/15
30% OF + 70% CF	C/30
45% OF + 55% CF	C/45
100% WF	W/100
15% OF + 85% WF	W/15
30% OF + 70% WF	W/30
45% OF + 55% WF	W/45

CF, corn flour; OF, oak flour; WF, wheat flour.

### 2.4 Analysis of flour types

Moisture, fat, protein, ash, fiber, and pH of flour types were measured according to AACC (2000) methods.

### 2.5 Analysis of biscuit samples

#### 2.5.1 Determination of peroxide value

The lipids of grinded biscuits (50 g) were extracted according to the method given by Mildner‐Szkudlarz et al. (2009) with some modifications. The extraction was done by using hexane solvent in a laboratory shaker in ambient conditions. After filtration and separation of lipid fraction, solvent was removed by evaporation under vacuum condition onto the rotary evaporator (Bokhi, Switzerland). The extracted oil was immediately used to calculate the peroxide value (PV) according to AOAC (1990) methods.

#### 2.5.2 Determination of antioxidant activity

The antioxidant activity of biscuit samples was measured in terms of radical scavenging ability using the stable radical 2,2‐diphenyl‐1‐pykrylhydrazyl (DPPH). One gram of the samples was mixed with 9 ml of ethanol solution (50%) and stirred for a minute. The samples were centrifuged for 10 min at 25°C and 203 ×g. One ml of 1 mmol/L DPPH methanolic solution was added to 3 ml of the supernatant phase and the resulting mixture was vigorously stirred. The mixture was left for 30 min in the dark at room temperature. The absorbance was measured at 517 nm. The radical scavenging activity was expressed as the inhibition percentage of free radical by the sample and was calculated using the following formula (Salmanian, Sadeghi Mahoonak, Alami, & Ghorbani, 2014):

$$\%\;\text{inhibition}=[\left(A_{0}-A_{t}\right)/A_{0}\times 100]$$

where *A*_*0*_ is the absorbance of the control (blank, without sample) and *A*_*t*_ is the absorbance of sample.

#### 2.5.3 Sensory evaluation

A five‐point hedonic scale was employed to evaluate the biscuit samples. A panel of 20 trained (for scoring of products and filling out the Questionnaire) naïve consumers and 20 food science and technology students evaluated the samples for taste, texture, aftertaste, color, and overall acceptance. The value scales ranged from 5 (*like extremely*) to 1 (*dislike extremely*) for each sensory property (Majzoobi, Hedayati, Habibi, Ghiasi, & Farahnaky, 2014).

### 2.6 Statistical analysis

All tests were conducted in a completely randomized design with three replications using the SAS 9.0 software (SAS Institute Inc., Cary, NC). All statistical comparisons were performed using the LSD test at the level of 5%.

## 3 RESULTS AND DISCUSSION

### 3.1 Chemical analysis of flour types

The flour types used were analyzed for pH, moisture, protein, fat, ash, and fiber contents. The results are shown in Table 3. An important factor influencing the overall quality (color, flavor, texture) is the pH (Sabrin, 2009). A comparison between the pH of these three flour types showed that CF had significantly (*p* < .05) higher pH (6.26) than WF or OF. The pH of WF and OF was the same (5.77), that is, they are more acidic than CF. OF had the lowest moisture (6.46%) and protein (4.14%) contents in comparison to CF and WF, while the highest fat (7.70%) and ash (1.83%) contents were also related to this flour type. There was a significant difference (*p* < .05) between flour types for these factors. Also, OF fiber content (2.51%) was significantly (*p* < .05) lower than that of WF (5.06%). In general, the differences between chemical components of the different flour types may be due to geographical location, the culture, and climatic conditions. Sabrin (2009) introduced acorns as a calorie‐dense food source because of the high levels of fat. Fat content can affect staling, so high‐fat flour types are best suited for use in low‐moisture foodstuff such as cookies or biscuits, because moisture can increase lipid oxidation unless antioxidants are included in the formulation. Also, according to Silva et al. (2016), OFs exhibit relevant amounts of lipids (8%–14%), low protein content (4%–5%), and 1.8%–2.1% ash content, which are in close agreement with our results.

**Table 3:** Chemical analysis of wheat, corn, and oak flour

	Flour type		
	Wheat	Corn	Oak
Moisture (%)	10.78 ± 0.08^a^	8.87 ± 0.07^b^	6.46 ± 0.07^c^
Protein (%)	11.13 ± 0.03^a^	6.19 ± 0.03^b^	4.14 ± 0.00^c^
Fat (%)	1.20 ± 0.01^b^	0.73 ± 0.02^c^	7.70 ± 0.00^a^
Ash (%)	0.28 ± 0.13^b^	0.58 ± 0.2^b^	1.83 ± 0.17^a^
Fiber (%)	5.06 ± 0.04^a^	2.82 ± 0.24^b^	2.51 ± 0.30^b^
pH	5.77 ± 0.04^b^	6.26 ± 0.04^a^	5.77 ± 0.01^b^

Mean values ± SD (*n* = 3). Different superscript letters indicate significant differences (*p* < .05).

### 3.2 Peroxide value

Peroxide value of biscuit samples was measured during storage; the results are shown in Table 4. The C/45 sample had the lowest PV during storage, followed by W/45. Increasing the OF percentage in formulations showed lower PV, due to high levels of phenolic compounds in OF. The C/100 sample had significantly (*p* < .05) lower PV than the W/100 sample, which showed higher phenolic compound amount in CF. This is in agreement with the observed higher phenolic compounds in corn muffin in comparison to wheat muffin (Korus, Gumul, Krystyjan, Juszczak, & Korus, 2017; Soong, Tan, Leong, & Henry, 2014). Cereal grains are a good source of antioxidants, including vitamin E, folates, minerals (iron, zinc), trace elements (copper, selenium, and manganese), carotenoids, and phytic acid. The most abundant free phenolic acids in wheat are ferulic, vanillic, and ρ‐coumaric acids, while corn is rich in ferulic acid and carotenoid (Fardet, Rock, & Rémésy, 2008). According to the results presented in Table 5, PV has increased in all biscuits during storage and there is a significant difference (*p* < .05) between Day 1 and Day 28.

**Table 4:** Peroxide value (O_2_ meq/kg oil) of biscuit samples during the 28 days storage

Biscuit sample formulation	Time (day)		
	1	14	28
C/100: (100% CF)	0.95 ± 0.02^ef^	1.23 ± 0.02^c^	1.25 ± 0.02^c^
C/15: (15% OF + 85% CF)	0.87 ± 0.01^gh^	1.01 ± 0.01^e^	1.10 ± 0.05^d^
C/30: (30% OF + 70% CF)	0.75 ± 0.20^ij^	0.57 ± 0.01^k^	0.70 ± 0.01^j^
C/45: (45% OF + 55% CF)	0.35 ± 0.20^m^	0.39 ± 0.20^lm^	0.56 ± 0.02^k^
W/100: (100% WF)	0.82 ± 0.00^hi^	1.53 ± 0.02^a^	1.59 ± 0.03^a^
W/15: (15% OF + 85% WF)	0.90 ± 0.01^fg^	0.96 ± 0.01^ef^	1.33 ± 0.03^b^
W/30: (30% OF + 70% WF)	0.81 ± 0.00^hi^	0.78 ± 0.01^i^	0.89 ± 0.02^fg^
W/45: (45% OF + 55% WF)	0.42 ± 0.01^ij^	0.57 ± 0.02^k^	0.75 ± 0.03^ij^

CF, corn flour; OF, oak flour; WF, wheat flour.

Mean values ± SD (*n* = 3). Different superscript letters indicate significant differences (*p* < .05).

**Table 5:** The overall mean of peroxide value and antioxidant activity of biscuit samples (regardless of the type of biscuit) during the 28 days storage

Oxidation properties	Time (day)		
	1	14	28
Peroxide value (O_2_ meq/kg oil)	0.73 ± 0.04^b^	0.88 ± 0.07^ab^	1.02 ± 0.06^a^
Antioxidant activity (%)	21.74 ± 1.07^a^	14.51 ± 1.31^b^	12.56 ± 1.31^b^

Mean values ± SD (*n* = 24). Different superscript letters indicate significant differences (*p* < .05).

### 3.3 Antioxidant activity

According to Table 6, antioxidant activity of biscuits containing CF + OF increased by increasing the OF percentage that showed higher OF antioxidant activity than CF. Similarly, the antioxidant activity of biscuits with WF + OF increased steadily by increasing the OF percentage. The samples of C/45 and W/45 had the highest antioxidant activity in different days among all samples. Also, C/100 sample had higher antioxidant activity than W/100, which was in agreement with the PV results. There is a direct correlation between the antioxidant activity and the amount of phenolic compounds (Soong et al., 2014). The high antioxidant activity in OF is due to its various antioxidants, especially α‐ and γ‐tocopherols, tannins (Molavi, Keramat, & Raisee, 2015), and gallic, caffeic, and chlorogenic acids. According to results of Korus et al. (2017), this is most likely related to the large amounts of polyphenols and tannins in biscuits with the addition of acorn flour and in flour itself, because tannins have 15–30 times greater activity than other polyphenols (TEAC for acorn flour: 76 mmol/L Tx kg^−1^). The flavonoids present in OF are one of the most important natural phytochemical compounds. These compounds have a wide range of physiochemical and biological activities, including the scavenging of free radicals and antioxidant activities (Liu & Yao, 2007). The methanolic extract of oak phenolic compounds increases the oxidative stability of sunflower oil and is a stronger antioxidant than BHT (Ghaderi, Alami, Sadeghi, Azizi, & Ghorbani, 2012). By detecting the changes in PV, oak acorn aqueous extract was found to influence the stability of pork fat, which showed the antioxidant activity (Rakic, Povrenovic, Tesevic, Simic, & Maletic, 2006). Based on Table 5, the antioxidant activity of all biscuits decreased during storage. There was a significant difference (*p* < .05) between the antioxidant activity in first day and those on Days 14 and 28. Antioxidant activity did not show any significant difference (*p* < .05) on the 14th and 28th days.

**Table 6:** Antioxidant activity (%) of biscuit samples during the 28 days storage

Biscuit sample formulation	Time (day)		
	1	14	28
C/100: (100% CF)	19.55 ± 0.50^cde^	10.10 ± 1.50^ijk^	8.54 ± 1.70^ijk^
C/15: (15% OF + 85% CF)	18.12 ± 0.80^def^	11.18 ± 1.80^hijk^	9.90 ± 2.00^ijk^
C/30: (30% OF + 70% CF)	24.83 ± 2.00^abc^	13.90 ± 1.10^fghi^	11.30 ± 2.40^hijk^
C/45: (45% OF + 55% CF)	28.95 ± 1.70^a^	25.85 ± 1.90^ab^	24.35 ± 2.10^abc^
W/100: (100% WF)	16.38 ± 1.80^efgh^	8.00 ± 1.60^jk^	6.50 ± 1.60^k^
W/15: (15% OF + 85% WF)	17.09 ± 1.80^defg^	12.04 ± 1.90^ghijk^	8.98 ± 0.08^ijk^
W/30: (30% OF + 70% WF)	21.17 ± 2.00^bcde^	12.74 ± 1.70^fghij^	11.44 ± 1.20^hijk^
W/45: (45% OF + 55% WF)	27.88 ± 2.00^a^	22.37 ± 1.70^bcd^	19.50 ± 1.60^cde^

CF, corn flour; OF, oak flour; WF, wheat flour.

Mean values ± SD (*n* = 3). Different superscript letters indicate significant differences (*p* < .05).

### 3.4 Sensory evaluation

The panelists evaluated the organoleptic properties of biscuit samples; the results are presented in Table 7. There was no significant difference (*p* < .05) between different samples for flavor, texture, aftertaste, color, or overall acceptability. It showed that OF did not have any adverse effect on panelists’ evaluation, and samples with this flour were similar to control samples. Previous results showed that sensory evaluation of the cake samples made from acorn–wheat flour blends indicated that the sample with 5% acorn flour was quite similar to the control and had acceptable texture and sensory properties as compared to the control (Molavi et al., 2015). Our results disagreed with previous studies, that is, the lower scores of texture, color, flavor, and overall acceptance of wheat biscuits with the addition of acorn flour (Korus et al., 2017).

**Table 7:** Sensory evaluation of biscuit samples during the 28 days storage

Biscuit sample formulation	Sensory properties				
	Flavor	Texture	After taste	Color	Overall acceptability
C/100: (100% CF)	4.20 ± 0.70^a^	4.20 ± 0.70^a^	4.05 ± 0.76^a^	4.00 ± 0.72^a^	4.10 ± 0.85^a^
C/15: (15% OF + 85% CF)	4.00 ± 0.72^a^	4.20 ± 0.62^a^	4.05 ± 0.76^a^	4.25 ± 0.64^a^	4.15 ± 0.59^a^
C/30: (30% OF + 70% CF)	4.05 ± 0.89^a^	4.10 ± 0.55^a^	4.00 ± 0.65^a^	4.10 ± 0.72^a^	4.00 ± 0.56^a^
C/45: (45% OF + 55% CF)	4.15 ± 0.74^a^	4.10 ± 0.85^a^	3.95 ± 0.82^a^	4.30 ± 0.92^a^	4.05 ± 0.69^a^
W/100: (100% WF)	3.95 ± 0.89^a^	4.05 ± 0.69^a^	3.60 ± 0.88^a^	4.15 ± 0.67^a^	4.00 ± 0.79^a^
W/15: (15% OF + 85% WF)	4.05 ± 0.83^a^	4.15 ± 0.67^a^	3.95 ± 0.76^a^	3.80 ± 0.83^a^	4.00 ± 0.79^a^
W/30: (30% OF + 70% WF)	3.95 ± 0.69^a^	4.10 ± 0.64^a^	3.70 ± 0.73^a^	3.80 ± 0.77^a^	4.05 ± 0.51^a^
W/45: (45% OF + 55% WF)	4.00 ± 0.79^a^	3.90 ± 0.79^a^	3.95 ± 0.69^a^	3.85 ± 0.59^a^	4.05 ± 0.60^a^

CF, corn flour; OF, oak flour; WF, wheat flour.

Mean values ± SD (*n* = 20). Different superscript letters indicate significant differences (*p* < .05).

## 4 CONCLUSION

The replacement of WF or CF with OF in biscuit formulations caused the biscuits nutritional value to increase. According to the results, the samples of C/45 and W/45 had higher antioxidant activity and lower peroxide value in comparison to other formulations. Also, samples containing CF showed higher antioxidant activities than formulations with WF. The sensory evaluation indicated no adverse effect on organoleptic properties, and the various OF percentages were the same. Therefore, this study proved that OF substitution could be useful for producing functional biscuits with higher antioxidant activity and—as a result—longer shelf life.

## CONFLICT OF INTEREST

None declared.
